# Model Predictive Control (MPC) of a Countercurrent Flow Plate Heat Exchanger in a Virtual Environment

**DOI:** 10.3390/s24144511

**Published:** 2024-07-12

**Authors:** Jairo Siza, Jacqueline Llanos, Paola Velasco, Alexander Paul Moya, Henry Sumba

**Affiliations:** 1 Department of Electrical, Electronic and Telecomunications, Universidad de las Fuerzas Armadas ESPE, Av. Gral. Rumiñahui s/n, Sangolquí 171103, Ecuador; pmvelasco1@espe.edu.ec; 2 Research Group of Propagation, Electronic Control, and Networking (PROCONET), Universidad de las Fuerzas Armadas ESPE, Av. Gral. Rumiñahui s/n, Sangolquí 171103, Ecuador; 3 Department of Engineering, Technology and Mathematics, Universidad Internacional de La Rioja (UNIR), Av. De la República E7-123 y pasaje Martín Carrión, Quito 170518, Ecuador

**Keywords:** inverse plant control, MPC control, virtualization, countercurrent flow plate heat exchanger, process control

## Abstract

This research proposes advanced model-based control strategies for a countercurrent flow plate heat exchanger in a virtual environment. A virtual environment with visual and auditory effects is designed, which requires a mathematical model describing the real dynamics of the process; this allows parallel fluid movement in different directions with hot and cold temperatures at the outlet, incorporating control monitoring interfaces as communication links between the virtual heat exchanger and control applications. A multivariable and non-linear process like the plate and countercurrent flow heat exchanger requires analysis in the controller design; therefore, this work proposes and compares two control strategies to identify the best-performing one. The first controller is based on the inverse model of the plant, with linear algebra techniques and numerical methods; the second controller is a model predictive control (MPC), which presents optimal control actions that minimize the steady-state errors and aggressive variations in the actuators, respecting the temperature constraints and the operating limits, incorporating a predictive model of the plant. The controllers are tested for different setpoint changes and disturbances, determining that they are not overshot and that the MPC controller has the shortest settling time and lowest steady-state error.

## 1. Introduction

Industrialization and production have increased considerably in different areas of the world, which leads to the existence of multiple variables that must be controlled correctly. Industrialization requires efficiency, precision, and quality in manufactured products, which means that labor is a limiting resource for industrial development, so implementing an automatic control that meets needs, saves time, and solves issues of non-linearity or multivariable process controls is required [1].

In a world with extensive pollution, the search for solutions to this problem has become a priority. Among the considerable sources of pollution are greenhouse gasses (GHGs) caused by excess waste heat, which is why plate heat exchangers (PHEs) emerge as a tool to mitigate pollution. The European Union (EU) currently estimates that energy use will increase by 32.5% by 2030 [2]. One relevant option is to optimize thermal energy recovery with the use of efficient and compact PHEs.

Because of the increase in energy demand and GHG emissions, efficient heat transfer management is necessary. A solution to these problems is to analyze the thermo-hydraulic performance of high-performance heat transfer systems [3,4]. Industries have used traditional heat exchangers such as shell and tube heat exchangers that reduce heat to 50%. These exchangers have characteristics that are difficult to correct, such as a reduced hydraulic diameter and temperature differences. Contrary to conventional heat exchangers, plate heat exchangers are more energy efficient and compact, in addition to their high-quality manufacturing, which allows them to support high temperatures and pressures, with heat restoration rates around 90% [5,6].

At the beginning of 1923, plate heat exchangers (PHEs) were developed in the industrial area due to their mechanical structural shape, solid design, resistant characteristics, capacity to resist pressure drops, and efficient heat transfer management [7]. Over time, these systems have become an essential part of the consumer, pharmaceutical, chemical, oil refining, and energy industries. In the same way, the modern energy sector has generated great interest in the study of various applications for efficient heating networks and low-temperature systems [8]. According to [9], this type of unit has increased utility value because industries presently have a great demand in relation to heat transfer efficiency, and a considerable reduction in energy consumption is needed.

The heat exchanger is used in different applications requiring heat transfer, and for this reason, there are different types of designs. In the oil and gas industry, they can have configurations with sealed plates, circuit boards, shell and tube (sensors), plate and countercurrent flow (PHE), and shell and tube (STHE) systems [10]. In chemical process applications, as well as refrigeration and heat recovery systems, shell and helical tube (HE)-type heat exchangers consisting of curved tubes inside a shell are frequently used. HE is used to reduce the temperature of lubricating oil [11]. Meanwhile, the scraped surface heat exchanger (SSHE) is used for ice slurry production and food processing. Exchangers also have applications in buildings with energy-efficient technologies [12].

Currently, plate heat exchangers (PHEs) are very popular due to their compactness and high heat transfer coefficient. They constitute the beginning of a very efficient heat recovery system since their high performance, in combination with countercurrent flow, can obtain temperatures close to the final values, which is the temperature of the air in the ventilated space [13]. They are most used in industrial applications, such as food, beverage, chemical, gas, and oil processes, among others, and are of interest for experimental, theoretical, and computational research.

Plate heat exchangers are extensively used in various industrial sectors, slightly less than the use of shell and tube heat exchangers [14]. Another aspect of PHEs is their compact size, as they take up less space, making them attractive for food industries [15]. PHEs are known for their high heat transfer efficiency in addition to their high thermal performance. There are variants of PHEs, such as corrugated plates, to strengthen the plates and produce turbulence in hot and cold fluids [16].

Industry 4.0, in areas of the industrialization of distillation processes, has been modernized in terms of control, i.e., shifting from conventional controllers to advanced controllers [17]. Due to their nature and complexity, PHEs present a great challenge in the area of control and supervision due to their distribution of transport parameters and variable time requirements, leading to steady-state studies because only contour measurements are available. For this reason, a simplified model containing the essential dynamics for the development is applied in this work. In [18], a number of applications in industry and engineering, such as energy production and chemical processes, among others, use two substances at different temperatures to exchange heat. The integrated energy system (IES) requires a link between an energy source and thermal loads. It is, therefore, essential to incorporate advanced controllers that reduce energy loss and ensure proper heat supply.

When the integral time is smaller, the error tends to decrease faster, but the system becomes underdamped if it is reduced too far, and the system becomes unstable [19]. The proportional–integral (PI) controller is slower, and the response period is 50% longer than that of the proportional control.

For the control of plate heat exchangers, traditional proportional integral derivative (PID) controls have been reported in [20], which present a very high response rate but low control. In [21], a PI control is shown where the system tends to be damped and unstable, and its response time is slower. However, it has been evidenced that, in highly non-linear, multivariable processes, traditional control algorithms such as PI and PID do not show the best performance in a transitory state; for example, in multivariable processes such as two-phase separators [22], three-phase separators and Desalination Plants [23], and combined cycle generation plants [24], advanced controllers have been demonstrated to be superior to traditional ones in aspects of overshoot, settling time, and rise time. 

Therefore, studies of PHEs have been presented that analyze the model and the non-linear parameters of this process, in order to develop optimal control algorithms. In the literature, there is limited information about the control of PHE. The control is applied to the output temperature according to [21] and uses an anticipatory control, focusing on the fuzzy model with a predictive control, in addition to classical PI and PID controllers with the Ziegler Nichols and Cohen Coon tuning methods. The other manuscript [25] describes a functional predictive control method based on a fuzzy model of the process and the prediction is performed with the Takagi–Sugeno (TS) technique. MPC controllers have been used in approximately 2500 industrial applications, such as the food, chemical, paper, refining, and petrochemical industries [26]. In [27], a model-based control of compact heat exchangers is proposed that allows the performance of heat transfer and its robustness to disturbances in the refrigerant distribution system. This controller uses the total terminal energy as the main control variable instead of the output temperature, while in [19], two control methods are applied: the first one is a linear Gaussian control (LQG) and is compared with quantitative feedback (QFT). These controllers are based on the temperature control of the cold stream at the PHE output, and these controllers consider the analysis of the effect of uncertainty in the parameters, variable setpoint changes, and error rates. In [28], a control of district heating processes is shown, which seeks the efficiency and stability of the heating substation, where a loop configuration controller with two degrees of freedom is presented and is compared with a PI control that rejects existing perturbations.

On the other hand, process virtualization has become an essential tool in the industrial field, facilitating that equipment, machinery, equipment, and plants are moved to an animated environment as a cyber-physical object. In industry, it is feasible to perform tests, configurations, programming, and prediction of maintenance virtually before application [29]. Virtualization consists of separating physical hardware from the execution of software, in order to have a greater operational versatility [30]. In different areas of industries such as the aerospace, defense, and automotive industries, industrial process model designs have been used to reduce time in the development, design, and validation of equipment, as well as to reduce manufacturing costs [31]. In the academic area, virtual environments similar to real production processes have been developed for control applications, for example, in [32], which shows a virtual generation plant based on solar collectors with real information from the Almeria plant in Spain, or virtual laboratories designed based on real laboratory stations, such as the virtualization of the FESTO reactor station [33].

This research proposes the development of controllers for a plate heat exchanger and countercurrent flow, which controls the variables of cold outlet temperature and hot outlet temperature; for this process, two types of advanced controls are applied to determine the most efficient and best performance in a transient and steady state in PHE. The first controller is based on linear algebra techniques, specifically based on the inverse model of the plant, and the second advanced controller is an MPC. The prediction model is based on differential equations, and the design of the controller provides robustness with respect to disturbances in the measurements of the control loop sensors. The mathematical model and the implemented controls are made through Matlab R2020a software; taking into account regulation tasks, an interactive environment with the user is considered, such as a virtual 3D environment of an industrial process, which is oriented to teaching and learning within the area of process control. This allows a quasi-real immersion through the simulation of the process with the use of the visual and sound effects of an industrial environment. The virtual development is made possible by the Unity 3D platform that together with the Visual Studio 2019 software allows the implementation of lines of code in which the models, effects, and communication links are added. The proposed controllers are finally validated for their performance in different scenarios that the plant may present in the event of a perturbation.

The main contribution of this research is the creation of an immersive virtual environment for a more intuitive interaction between the user and the industrial process, allowing the adaptation and identification of instruments, subprocesses, process variables, etc., and also having the possibility to manipulate different parameters and experiment with different configurations before being taken to the real plant. For a correct operation, two controllers are implemented and compared in order to visualize their efficiency in the process. The first controller focuses on the principles of linear algebra-denominated control based on the inverse of the plant, which requires knowledge of the behavior of the plant and the complications with non-linear systems or with uncertainties; therefore, an advanced control technique (MPC) ideal for non-linear systems is proposed to predict the behavior of the system in the future. The model considers the structure of the PHE and the ideal conditions of the system, with the objective of applying the corresponding modeling techniques and starting from the energy balance principle in order to obtain the differential equations that describe the system.

## 2. Description of the Counter Current Flow Plate Heat Exchanger

**Figure 1:**
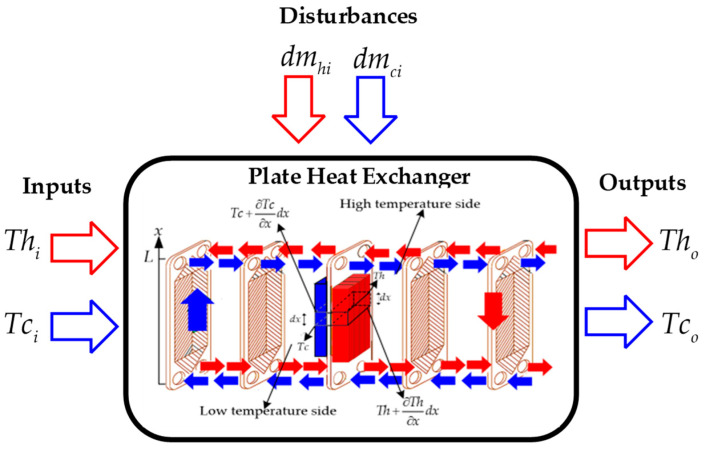
Countercurrent flow plate heat exchanger developed from authors.

The heat exchanger is structured by a number of metal plates with parallel corrugations providing the direction of movement to the fluids that propagate in these channels. These plates are bolted together and covered with two covers. Each plate has four holes that allow the circulation of water in parallel but in different directions. The available water is at different temperatures—hot water and cold water. The hot water is driven by the even plates at the same time the cold water is driven by the odd plates so that there is heat exchange between the two fluids as shown in Figure 1 [28].

**Figure 2:**
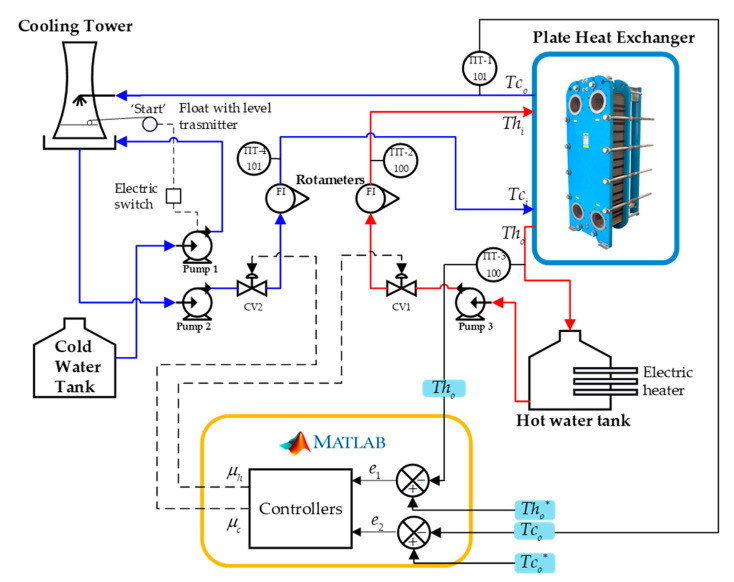
P&ID diagram of the industrial process of a parallel plate heat exchanger with countercurrent flow.

The P&ID diagram in Figure 2 describes the operation of the process, connected to the parallel plate heat exchanger and countercurrent flow. The process presents a control loop 100 corresponding to the hot water line (red arrow), while the cold water (blue arrow) belongs to the control loop 101. The temperatures are controlled by the variables’ $\left(\mu_{h}\right)$ and $\left(\mu_{c}\right)$ signals that go to the control valves $\left(CV_{1}\right)$ and $\left(CV_{2}\right)$ for each line of the process.

The control loop 100 presents the reading of the hot output temperature $\left(Th_{o}\right)$ through a temperature-indicating transmitter $\left(TIT3-100\right)$ whose signal enters a comparator where it is subtracted from the desired hot output temperature $\left({{Th}_{o}}^{*}\right)$. The difference generates an error $\left(e_{1}\right)$ that enters the controller and generates the control action $\left(\mu_{h}\right)$ that is directed to the control valve $\left(CV_{1}\right)$ of the hot fluid line 100, regulating the flow from a tank of hot water. In the same way, the control loop 101 contains the reading of the cold outlet temperature $\left(Tc_{o}\right)$ through a temperature-indicating transmitter $\left(TIT1-101\right)$ which generates a signal to the second comparator which is subtracted from the desired cold outlet temperature $\left({{Tc}_{o}}^{*}\right)$. The difference generates an error $\left(e_{2}\right)$ which is corrected by the control action $\left(\mu_{c}\right)$ that goes to the control valve $\left(CV_{2}\right)$ of the cold fluid line 101, where the flow from a cooling tower is regulated. The cooling tower is supplied by a cold water tank when the liquid level in the cooling tower is low, so a pump is turned on until a considerable liquid level is reached in the cooling tower.

For control applications, the following variables are determined as variables to be controlled: hot output temperature $\left(Th_{o}\right)$ and cold output temperature $\left(Tc_{o}\right)$, and as manipulated variables: hot input temperature $\left(Th_{i}\right)$ and cold input temperature $\left(Tc_{i}\right)$.

### 2.1. Mathematical Modeling of the Countercurrent Flow Parallel Plate Heat Exchanger

In the development of the mathematical modeling of the counter-flow plate heat exchanger, different aspects of the structure and the thermodynamic process that takes place inside the heat exchanger are considered. These considerations previous to the mathematical modeling of the plate heat exchanger and countercurrent flow are as follows: (a) Under ideal and static operating conditions, i.e., the rates of change in flow $\left(\Delta m\right)$ and temperature $\left(\Delta T\right)$ are constant and independent of time only at the time of modeling; (b) Heat losses to or from the environment are negligible; (c) There are no sources of thermal energy in the exchanger walls; (d) The temperature of each fluid is uniform over each cross-section of the exchanger; (e) There is no phase change in the fluid stream; (f) Longitudinal heat conduction between the fluids and the walls is negligible; (g) The heat transfer coefficients $\left(U\right)$ are constant; (h) The specific heat $\left(C_{i}\right)$of each fluid is constant; (i) The heat transfer surface is uniformly distributed on each side of the fluid [18,34].

Let $\left(T\right)$ be a function of temperature depending on time $\left(t\right)$ and the mass flow of the fluid as seen in Equation (1):

(1) $$T=f\left(m,t\right)$$

What is transferred in the plates at that instant is energy, so the accumulated energy $\left(E_{a}\right)$ is the sum of the input energy flow $\left(E_{i}\right)$ minus the output energy flow $\left(E_{o}\right)$ plus the gained energy flow $\left(E_{g}\right)$ minus the lost energy flow ($E_{l}$), obtaining (2).

(2) $$E_{a}=E_{i}-E_{o}+E_{g}-E_{l}$$

If the energy balance is performed at the plate side, then the temperature flux is constant. According to this characteristic, the energy flow lost $\left(E_{l}\right)$ is equal to zero. In addition, this balance is performed on the cold junction plate. If we take into account the direction of flow of the accumulated energy and the time instant $\left(\Delta t\right)$ over the longitudinal area of the plate, subjected to a delta of the energy gained, we obtain (3):

(3) $$\frac{\Delta E_{a}}{\Delta t}={\dot{E}}_{i}-{\dot{E}}_{o}+{\dot{E}}_{g}$$

where (3) is defined by the increase in the cumulative temperature around the plate $\left(\frac{\Delta E_{a}}{\Delta t}=\frac{E_{t_{1}}-E_{t_{0}}}{t_{1}-t_{0}}\right)$, which is equal to the increase in the rate of energy flow at the inlet $\left({\dot{E}}_{i}\right)$minus the increase in the rate of energy flow at the outlet $\left({\dot{E}}_{o}\right)$ and the increase in the rate of energy gained $\left({\dot{E}}_{g}\right)$.

In this section, the energy equation is based on the dynamic and physical principles. Where the energy $\left(E\right)$ is equal to the sum of the internal energies of the plate $\left(\theta_{itl}\right)$, kinetic $\left(k\right)$, and potential $\left(\varphi \right)$, we obtain Equation (4).

(4) $$E=\theta_{itl}+k+\varphi$$

As defined at the beginning, the plate and countercurrent flow exchanger are not taken in a reference frame with respect to the speeds of both the fluid flow and the exchanger itself, and there is no reference frame with respect to the height of the kinetic energies $\left(k\right)$ and potential $\left(\varphi \right)$, i.e., these variables are equal to zero, resulting in Equation (5).

(5) $$E=\theta_{itl}$$

The internal energy $\left(\theta_{itl}\right)$ is composed of the product between the mass of the fluid or mass flow rate $\left(m\right)$ and the specific heat with respect to the volume of the fluid $\left(C_{v}\right)$ and the difference between the average temperature $\left(T\right)$ and a reference temperature $\left(T_{R}\right)$, obtaining (6).

(6) $$\theta_{itl}=m\cdot C_{v}\left(T-T_{R}\right)$$

The total differential of the internal energy $\left(d\theta_{itl}\right)$ is equal to the addition of the partial derivative of the same internal energy with respect to temperature $\left(dT\right)$ at a constant volume and the partial derivative of the same internal energy with respect to volume $\left(dV\right)$ at a constant temperature, as shown in Equation (7).

(7) $$d\theta_{itl}={\left(\frac{\partial \theta_{itl}}{\partial T}\right)}_{V}dT+{\left(\frac{\partial \theta_{itl}}{\partial V}\right)}_{T}dV$$

Also, the specific heat with respect to volume $\left(C_{v}\right)$ is equal to the partial derivative of the internal energy with respect to temperature at a constant volume and its expression is given in (8).

(8) $${\left(\frac{\partial \theta_{itl}}{\partial T}\right)}_{V}dT=C_{v}$$

It is considered that the partial derivative of the internal energy, with respect to temperature at a constant volume, $\left({\left(\frac{\partial \theta_{itl}}{\partial T}\right)}_{V}dT\right)$ approaches the partial derivative of the enthalpy $\left(H\right)$ with respect to temperature at a constant pressure $\left({\left(\frac{\partial H}{\partial T}\right)}_{P}dT\right)$, obtaining the specific heat with respect to pressure $\left(C_{p}\right)$. As indicated in the conditions, these parameters remain constant for incompressible liquids and solids, thus defining Equation (9).

$${\left(\frac{\partial \theta_{itl}}{\partial T}\right)}_{V}dT\approx {\left(\frac{\partial H}{\partial T}\right)}_{P}dT$$

$${\left(\frac{\partial \theta_{itl}}{\partial T}\right)}_{V}\approx {\left(\frac{\partial H}{\partial T}\right)}_{P}$$

(9) $$C_{v}=C_{p}=C$$

Equations (6) and (7) are replaced in (5) in terms of mass flow increments and give (10).

(10) $$\dot{E}=\dot{m}\cdot C_{p}\left(T-T_{R}\right)$$

Substituting (10) into (3), we obtain the generalized outlet temperature for any mass flow rate represented in (11).

(11) $$\frac{\Delta \left(mC_{p}T\right)}{\Delta t}={\dot{m}}_{i}\cdot C_{p}\cdot T_{i}-{\dot{m}}_{o}\cdot C_{p}\cdot T_{o}+{\dot{E}}_{g}$$

We posit that the energy gain $\left(E_{g}\right)$ is also a type of internal energy for the plate in which the model is analyzed and is related to the heat in the longitudinal area of the plate $\left(L\right)$ plus the work $\left(W\right)$. Above, it is specified that there is no energy variation from and to the surroundings; then, the work is equal to zero as observed in (12).

$${\dot{E}}_{g}=\dot{\theta }=\dot{L}+\dot{W}$$

(12) $${\dot{E}}_{g}=\dot{L}$$

Equation (11) is rewritten according to the above considerations, thus obtaining (12), and the increments are related to the mass flows $\left({\dot{m}}_{i}\right)$ and $\left({\dot{m}}_{o}\right)$, obtaining Equation (13).

(13) $$\frac{\Delta \left(mC_{p}T\right)}{\Delta t}={\dot{m}}_{i}\cdot C_{p}\cdot T_{i}-{\dot{m}}_{o}\cdot C_{p}\cdot T_{o}+\dot{L}$$

The first law of thermodynamics indicates that the rate of heat transfer from the hot fluid $\left(Q_{h}\right)$ is equal to the heat transfer to the cold fluid $\left(Q_{c}\right)$ as seen in Equations (14) and (15), keeping the increments for the modeling process as follows:

(14) $$Q_{h}={\dot{m}}_{h}\cdot C_{ph}\left(Th_{o}-Th_{i}\right)$$

(15) $$Q_{c}={\dot{m}}_{c}\cdot C_{pc}\left(Tc_{o}-Tc_{i}\right)$$

where $\left({\dot{m}}_{h}\right)$ and $\left({\dot{m}}_{c}\right)$ are the hot and cold mass flow increments, $\left(C_{ph}\right)$ and $\left(C_{pc}\right)$ are the specific heats of the hot and cold fluids, $\left(Th_{i}\right)$ and $\left(Tc_{i}\right)$ are the inlet temperatures of the hot and cold fluids, and $\left(Th_{o}\right)$ and $\left(Tc_{o}\right)$ are the outlet temperatures of the hot and cold fluids, respectively.

The hot stream produces a heat flow that must overcome the overall resistance (1/U⋅A) to transfer heat in order to obtain a temperature rise of the cold stream. Where (A) is the heat transfer area, (U) is the heat transfer coefficient, and ΔT is the temperature difference, the total heat transferred $\left(Q_{t}\right)$ is expressed according to Equation (16).

(16) $$Q_{t}=A\cdot U\cdot \Delta T$$

We designate ΔT as the difference between the cold (Tc) and hot (Th) temperatures. (U) presents a perturbation because it depends on the temperatures, characteristics, and velocity of the flow. The total heat transfer area is represented by Equation (17) where $\left(N_{p}\right)$ is the number of plates, and $A_{p}$ is the plate area.

(17) $$A=N_{p}A_{p}$$

The overall heat transfer coefficient (U) can be expressed according to Equation (18), where $\left(\lambda_{h}\right)$ is the convective heat transfer coefficient of the hot fluid, $\left(\lambda_{c}\right)$ is the convective heat transfer coefficient of the cold fluid, $\left(j_{p}\right)$ is the plate thickness, $\left(z_{p}\right)$ is the thermal conductivity of the plate, $\left(q_{f,c}\right)$ is the fouling factor of the cold fluid, and $\left(q_{f,h}\right)$ is the fouling factor of the hot fluid.

(18) $$U=\frac{1}{\frac{1}{\lambda_{h}}+\frac{j_{p}}{z_{p}}+\frac{1}{\lambda_{c}}+q_{f,c}+q_{f,h}}$$

An important consideration is the convective heat transfer coefficient (λ) which is subject to fluid properties, velocity, and plate geometry. This coefficient allows the calculation of the most important parameter which is the total heat transfer inside the plate heat exchanger. For the design of the PHE, the log mean temperature difference method is used. For this method, the heat transfer rate is given by Equation (19):

(19) $$Q_{t}=UA(F\Delta T_{med})$$

where $\left(T_{med}\right)$ is the logarithmic mean temperature difference expressed in Equation (20). (F) is the correction factor for the logarithmic mean temperature difference.

(20) $$\Delta T_{med}=\frac{\Delta T_{1}-\Delta T_{2}}{In(\Delta T_{1}/\Delta T_{2})}$$

The following conditions must be considered for the case of countercurrent flow.

$$\begin{array}{l} \Delta T_{1}=\left\{Th_{i}-\right.Tc_{o}\\ \Delta T_{2}=\left\{Th_{o}-\right.Tc_{i} \end{array}$$

The correction factor is a function of the heat exchanger configuration and dimensionless parameters such as the heat capacity coefficient (R) and the temperature efficiency (Pc). For the case of countercurrent flow devices, the correction factor is equal to one. Also, the end channels of the PHE only exchange heat with one adjacent channel; therefore, the countercurrent or concurrent flow is equal to one in two extreme situations as presented in Equations (21) and (22).

(21) $$R=\frac{Th_{i}-Th_{o}}{Tc_{o}-Tc_{i}}=\frac{{\left(mC_{p}\right)}_{c}}{{\left(mC_{p}\right)}_{h}}$$

(22) $$Pc=\frac{Tc_{o}-Tc_{i}}{Th_{o}-Th_{i}}=\frac{\Delta Tc}{\Delta Th}$$

Equation (23) represents the rate of energy stored inside the heat exchanger $\left({\dot{Q}}_{r}\right)$ and is defined as the difference between the thermal energy provided by the flow $\left({\dot{Q}}_{y}\right)$ and the total heat transfer $\left({\dot{Q}}_{t}\right)$.

(23) $${\dot{Q}}_{r}={\dot{Q}}_{y}-{\dot{Q}}_{t}$$

While ${\dot{Q}}_{r}=\rho \cdot v\cdot T$ is given by the product between the density of water ρ and the fluid velocity (*v*) and the temperature (T). Equations (14)–(16) and (23) allow us to determine the energy balance for each current as expressed in Equations (24) and (25):

(24) $$M_{h}\cdot C_{ph}\cdot \dot{T}h_{o}=m_{h}\cdot C_{ph}\left(Th_{i}-Th_{o}\right)-U\cdot A\left(Th_{o}-Tc_{o}\right)$$

(25) $$M_{c}\cdot C_{pc}\cdot \dot{T}c_{o}=m_{c}\cdot C_{pc}\left(Tc_{i}-Tc_{o}\right)-U\cdot A\left(Tc_{o}-Th_{o}\right)$$

where $M_{h}$ and $M_{c}$ are the masses of the hot and cold fluids, $C_{ph}$ and $C_{pc}$ are the specific heats of the hot and cold fluids, $Th_{i}$ and $Tc_{i}$ are the inlet temperatures of the hot and cold fluids, and $Th_{o}$ and $Tc_{o}$ are the outlet temperatures of the hot and cold fluids, respectively. The average temperature (Th) and (Tc) on the hot side and cold side are expressed in Equations (26) and (27).

(26) $$Th=\frac{Th_{i}+Th_{o}}{2}$$

(27) $$Tc=\frac{Tc_{i}+Tc_{o}}{2}$$

The energy balance in the steady state around the cold plate and hot plate is realized by implementing the equations of the mean temperatures of the hot and cold sides in Equations (28) and (29). These equations represent the non-linear mathematical model of the counter-flow plate heat exchanger.

(28) $$M_{h}\cdot C_{ph}\frac{dT_{ho}(t)}{dt}=m_{h}\cdot C_{ph}\left(Th_{i}-Th_{o}\right)+U(t)\cdot A\left[\frac{Th_{i}-Tc_{o}(t)}{2}+\frac{Th_{o}(t)-Tc_{i}}{2}\right]$$

(29) $$M_{c}\cdot C_{pc}\frac{dT_{co}(t)}{dt}=m_{c}\cdot C_{pc}\left(Tc_{i}-Tc_{o}\right)-U(t)\cdot A\left[\frac{Th_{i}-Tc_{o}(t)}{2}+\frac{Th_{o}(t)-Tc_{i}}{2}\right]$$

### 2.2. Virtual Environment Developed

**Figure 3:**
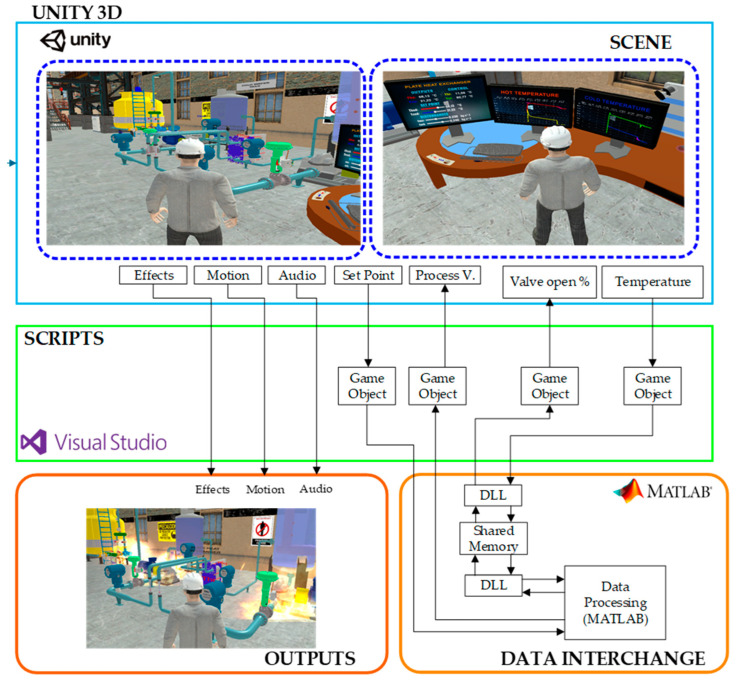
Virtualized system methodology of a counter-flow plate heat exchanger.

Figure 3 represents the virtualization process and connection between Unity Hub 3.7.0 software and Matlab R2020a software, where they work together in the process of a countercurrent flow plate heat exchanger. The Unity Hub 3.7.0 software allows for realistic virtualized environments in 2D and 3D planes, in addition to being suitable and compatible for control applications, in order to guide the community to increase their educational level in the industrial field since it shows a metaverse environment, which allows an immersive experience in education.

Figure 3 is made up of four sections, the light blue section corresponds to the scene, i.e., the virtual environment of the plate heat exchanger and countercurrent flow together with the control panels and visualization of the process variables. All these elements have been designed and modeled using a CAD platform, in this case AutoCAD Plant 3D and SketchUP. These designs are converted to .fbx format for later import into Unity Hub 3.7.0 software. The green section Scripts is composed of Game Objects which contain and organize components that define the behavior of the objects in a scene, besides allowing the exchange of data with the Matlab R2020a software, in addition to the digitization of the code that allows the exchange of information between Matlab and Unity 3D. The orange section contains the Matlab R2020a software where the mathematical model of the process is implemented, in addition to the control architectures. The communication is performed through shared memories through a DLL program which serves as a connection point between the Matlab R2020a software and Unity 3D. The red-colored section called output includes process operation effects such as sounds and alarms and the behavior in the event of a disturbance.

## 3. Design of System Control Strategies

**Figure 4:**
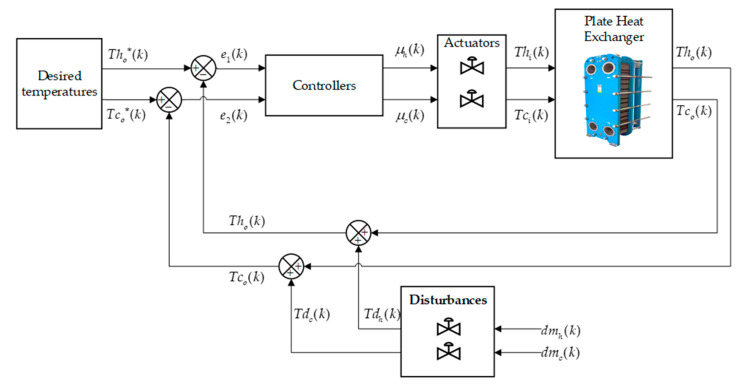
Closed-loop control scheme of the countercurrent flow plate heat exchanger.

Figure 4 shows the general closed-loop scheme of the plate heat exchanger, in which two control architectures are applied in order to compare and observe the desired dynamic behavior of the outlet temperature $\left(Tc_{o}\right)$ and $\left(Th_{o}\right)$, which changes due to the effect of the input of two temperatures, one cold and the other hot: $\left(Tc_{i}\right)$ and $\left(T_{hi}\right)$. The process is perturbed to evaluate the efficiency of each controller.

Figure 4 details the general control scheme, where the control signals leaving the controller are represented by the terms $\mu_{c}\left(k\right)$ and $\mu_{h}\left(k\right)$, and this signal enters the process, i.e., the input temperatures $T_{i}(k)$. Also, the term representing the desired temperatures are ${{Tc}_{o}}^{*}\left(k\right)$ and ${{Th}_{o}}^{*}\left(k\right)$ for regulation tasks, while the control errors are given by Equation (30). The output temperatures taking into account the perturbation is given in Equation (31):

(30) $$e_{1}\left(k\right)={{Tc}_{o}}^{*}\left(k\right)-Tc_{o}\left(k\right),\;e_{2}\left(k\right)={{Th}_{o}}^{*}\left(k\right)-Th_{o}\left(k\right)$$

(31) $$Th_{o}\left(k\right)=Th_{o}\left(k\right)+Td_{h}(k),\;Tc_{o}\left(k\right)=Tc_{o}\left(k\right)+Td_{c}(k)$$

where $dm_{c}(k)$, $dm_{h}(k)$ is a vector of disturbances in the control loop sensor measurements.

### 3.1. Control Based on the Inverse of the Plant

**Figure 5:**
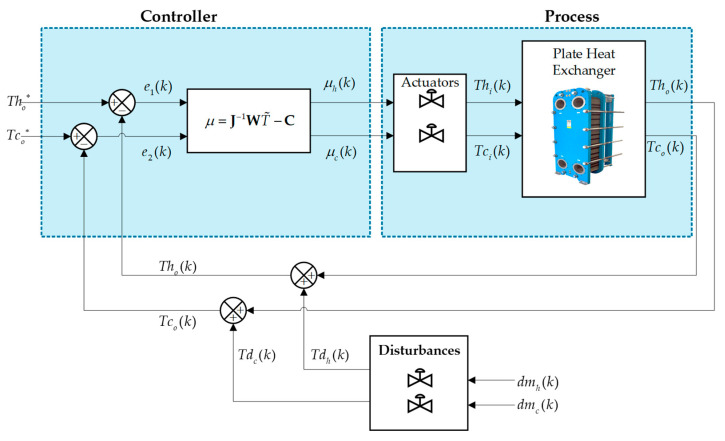
Inverse-based control scheme of the plate heat exchanger plant and countercurrent flow.

Figure 5 details the general diagram of the control based on the inverse of the plant. This controller, commonly known as non-linear internal model control, has evolved in applications in the industrial sector and its possible deconfiguration of the model [35]. The development of the controller is obtained by inverting the model, considering the development of differential equations describing the plant behavior and expressing it in matrix form $\dot{T}=WT_{i}+C$, where it represents the non-linear model of the process in matrix form. Therefore, the vectors $\dot{T}$ are the outputs of the process, W is the matrix associated with the dynamic parameters of the process, the vectors $T_{i}$ are the inputs to the process, and the matrix C is a matrix associated with the non-evolving states of the process.

The equations of the (32) and (33) model are represented in the continuous time domain.

(32) $$\frac{dTc_{o}}{dt}=\frac{A\cdot U(t)}{M_{c}\cdot C_{pc}}\left[\frac{Th_{i}-Tc_{o}(t)}{2}+\frac{Th_{o}(t)-Tc_{i}}{2}\right]+\frac{m_{c}}{M_{c}}\left(Tc_{i}-Tc_{o}\right)$$

(33) $$\frac{dTh_{o}}{dt}=-\frac{A\cdot U(t)}{M_{h}\cdot C_{ph}}\left[\frac{Th_{i}-Tc_{o}(t)}{2}+\frac{Th_{o}(t)-Tc_{i}}{2}\right]+\frac{m_{h}}{M_{h}}\left(Th_{i}-Th_{o}\right)$$

Matrix representation of the model, which will be implemented in the closed-loop control scheme.

$$\begin{array}{l} x=f\left(x,u\right)\\ \dot{x}=A\Delta x+B\Delta u\\ x=\left[\begin{array}{l} T_{co}\\ T_{ho} \end{array}\right]\\ u=\left[\begin{array}{l} T_{ci}\\ T_{hi}\\ m_{c}\\ m_{h} \end{array}\right] \end{array}$$

$$\dot{x}=\left[\begin{array}{cc} \frac{dT_{co}}{dT_{co}} & \frac{dT_{co}}{dT_{ho}}\\ \frac{dT_{ho}}{dT_{co}} & \frac{dT_{ho}}{dT_{ho}} \end{array}\right]\left[\begin{array}{c} \Delta T_{co}\\ \Delta T_{ho} \end{array}\right]+\left[\begin{array}{cccc} \frac{dT_{co}}{dT_{ci}} & \frac{dT_{co}}{dT_{hi}} & \frac{dT_{co}}{dm_{c}} & \frac{dT_{co}}{dm_{h}}\\ \frac{dT_{ho}}{dT_{ci}} & \frac{dT_{ho}}{dT_{hi}} & \frac{dT_{ho}}{dm_{c}} & \frac{dT_{ho}}{dm_{h}} \end{array}\right]\left[\begin{array}{c} \Delta T_{ci}\\ \Delta T_{hi}\\ \Delta m_{c}\\ \Delta m_{h} \end{array}\right]$$

$$\begin{array}{l} \left[\begin{array}{c} \dot{\Delta }T_{co}\\ \dot{\Delta }T_{ho} \end{array}\right]=\left[\begin{array}{cc} -\frac{AU}{2M_{c}C}-\frac{m_{ceq}}{M_{c}} & \frac{AU}{2M_{c}C}\\ \frac{AU}{2M_{h}C} & -\frac{AU}{2M_{h}C}-\frac{m_{heq}}{M_{h}} \end{array}\right]\left[\begin{array}{c} \Delta T_{co}\\ \Delta T_{ho} \end{array}\right]+...\\ \;\;\;\;\;\;\;\;\;\;...+\left[\begin{array}{cccc} -\frac{AU}{2M_{c}C}+\frac{m_{ceq}}{M_{c}} & \frac{AU}{2M_{c}C} & \frac{T_{cieq}-T_{coeq}}{M_{c}} & 0\\ \frac{AU}{2M_{h}C} & -\frac{AU}{2M_{h}C}+\frac{m_{heq}}{M_{h}} & 0 & \frac{T_{hieq}-T_{hoeq}}{M_{h}} \end{array}\right]\left[\begin{array}{c} \Delta T_{ci}\\ \Delta T_{hi}\\ \Delta m_{c}\\ \Delta m_{h} \end{array}\right] \end{array}$$

(34) $$\begin{array}{c} \left[\begin{array}{c} {\dot{T}}_{co}\\ {\dot{T}}_{ho} \end{array}\right]=\left[\begin{array}{cc} -\frac{AU}{2M_{c}C}+\frac{m_{c}}{C} & \frac{AU}{2M_{c}C}\\ \frac{AU}{2M_{h}C} & -\frac{AU}{2M_{h}C}+\frac{m_{h}}{M_{h}} \end{array}\right]\left[\begin{array}{c} T_{co}\\ T_{ho} \end{array}\right]+\left[\begin{array}{cc} -\frac{AU}{2M_{c}C}-\frac{m_{c}}{M_{c}} & \frac{AU}{2M_{c}C}\\ \frac{AU}{2M_{h}C} & -\frac{AU}{2M_{h}C}+\frac{m_{h}}{M_{h}} \end{array}\right]\left[\begin{array}{c} T_{ci}\\ T_{hi} \end{array}\right]\\ \dot{T}=JT+C \end{array}$$

Methodology for Controller Design

The process is of the following form:

$$\dot{T}=JT+C$$

(35) $$T=J^{-1}\dot{T}-C$$

Considering regulation tasks, we have the error expressed as

T˜=T∗−TTd=cteT˜˙=T∗−T˙

(36) $$\dot{T}=-\dot{\tilde{T}}$$

Substituting (36) in (35) and considering a weight matrix W to saturate the errors,

$$T=J^{-1}W\tilde{T}-C$$

According to Figure 5, the control actions are equal to the temperatures entering the process μ=T, and we have the control law in (37).

(37) $$\;\mu =J^{-1}W\tilde{T}-C$$

### 3.2. Predictive Control (MPC)

**Figure 6:**
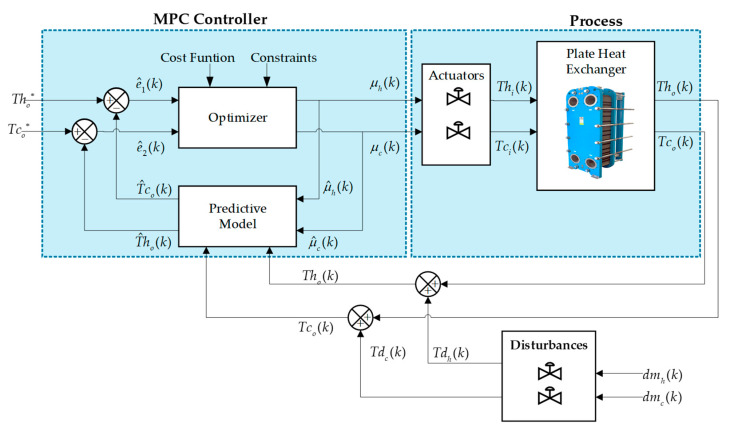
Closed-loop MPC control scheme of the countercurrent flow plate heat exchanger.

The model-based predictive control algorithm makes use of an internal dynamic prediction model of the process, a history of past information, an optimization cost function J, a prediction horizon, a control horizon, weights, and constraints associated with the process [36]. The mathematical model of the countercurrent plate heat exchanger is intended to predict the future behavior of its outputs: for this case, the cold outlet temperature $\hat{T}c_{o}$ and as the hot outlet temperature $\hat{T}h_{o}$ as a function of $Tc_{o}(k)$, $Th_{o}(k)$, $\mu_{c}(k)$, $\mu_{h}(k)$, present and past. This predicted output vector is subtracted from the vector of (SP) to obtain the errors ${\hat{e}}_{1}=\left({{Th}_{o}}^{*}-\hat{T}h_{o}(k)\right)$ and ${\hat{e}}_{2}=\left({{Tc}_{o}}^{*}-\hat{T}c_{o}(k)\right)$. The resulting control actions are subject to the weights (see Figure 6).

The MPC control includes an optimization problem where an objective function J(k) is posed which is presented in Equation (38) responsible for minimizing errors and abrupt control action changes. The first term ${\left[\hat{T}c_{o}(k+i|k)-{{Tc}_{o}}^{*}(k+i|k)\right]}^{2}$ minimizes the squared error of the cold temperature, which is multiplied by a weight denoted by $\gamma_{1}(k)$. To minimize the cold temperature error, the second term ${\left[\hat{T}h_{o}(k+i|k)-{{Th}_{o}}^{*}(k+i|k)\right]}^{2}$ with its assigned error $\gamma_{2}(k)$ is considered. In the controller formulation, one of the purposes is to protect the actuator. That is why an additional control objective is included. To minimize the changes in the control actions, therefore, we have ${\left[\Delta u_{1}\left(k+i-1\right)\right]}^{2}$, i.e., the square of the variation in the control actions for the input cold temperature variable with its respective weight $\rho_{1}(k)$. For the control variable hot entry temperature, we have its respective variation in the control action ${\left[\Delta u_{2}\left(k+i-1\right)\right]}^{2}$ and its respective weight $\rho_{2}(k)$, where k indicates a sample, $N_{p}$ is the prediction horizon, and $N_{c}$ is the control horizon.

(38) $$\begin{array}{c} J(k)=\sum_{i=0}^{N_{p}}\gamma_{1}(k){\left[\hat{T}c_{o}(k+i|k)-{{Tc}_{o}}^{*}(k+i|k)\right]}^{2}+\gamma_{2}(k){\left[\hat{T}h_{o}(k+i|k)-{{Th}_{o}}^{*}(k+i|k)\right]}^{2}+...\\ +...\sum_{i=0}^{N_{c}}\rho_{1}(k){\left[\Delta u_{1}\left(k+i-1\right)\right]}^{2}+\rho_{2}(k){\left[\Delta u_{2}\left(k+i-1\right)\right]}^{2} \end{array}$$

In general terms, $\hat{T}c_{o}(k+i|k)$ is the predicted cold outlet temperature, while $\hat{T}h_{o}(k+i|k)$ is the predicted hot outlet temperature. As ${{Tc}_{o}}^{*}(k+i|k)$ is the desired cold temperature, similarly, ${{Th}_{o}}^{*}(k+i|k)$ is the desired hot temperature. As for the variations in the control actions, the expression $\rho_{i}(k){\left[\Delta u_{2}\left(k+i-1\right)\right]}^{2}$ is obtained.

The countercurrent flow plate heat exchanger has operating constraints that are included in the optimization problem and are considered limits of the cold outlet temperature $Tc_{o}$ defined in Equation (39), where $Tc_{o\min}$ is the minimum cold outlet temperature, and $Tc_{o\max}$ is the maximum cold outlet temperature. The temperature described in Equation (40) represents the operating range of $Th_{o}$, where $Th_{o\min}$ is the minimum hot outlet temperature, and $Th_{o\max}$ is the maximum hot outlet temperature. As for the control value, its values were restricted between a maximum limit $\left(\Delta \mu_{\max}\right)$ and a minimum limit $\left(\Delta \mu_{\min}\right)$, as seen in Equation (41), and the second control action is represented in Equation (42).

(39) $$Tc_{o\min}\leq Tc_{o}(t)\leq Tc_{o\max}$$

(40) $$Th_{o\min}\leq Th_{o}(t)\leq Th_{o\max}$$

(41) $$\Delta \mu_{\min}\leq \Delta \mu_{1}\leq \Delta \mu_{\max}$$

(42) $$\Delta \mu_{\min}\leq \Delta \mu_{2}\leq \Delta \mu_{\max}$$

## 4. Results

This section shows the results of the interface designed in an immersive virtual environment: subsequently, the control strategies based on the inverse of the plant and MPC are compared in order to determine the best-performing control.

### 4.1. Virtual Countercurrent Flow Plate Heat Exchanger Environment

**Figure 7:**
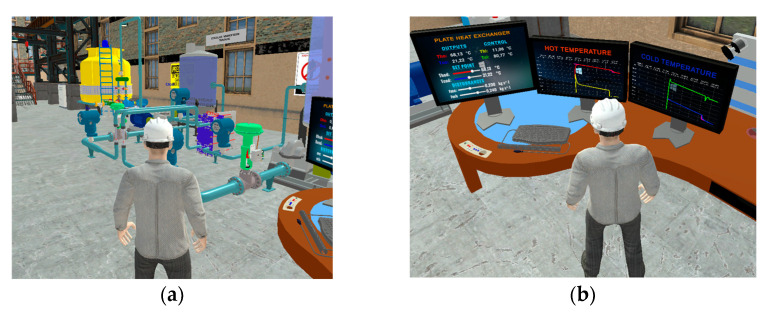
Virtual process environment: (**a**) industrial environment with heat exchanger and (**b**) monitoring and control area.

The application developed was tested on a computer with the following characteristics: 16 GB of RAM, 2.4 GHz processing speed, and a Ryzen 9 processor, generating a dynamic interaction with the user in a realistic environment. The interface is shown in Figure 7a with all the elements that make up the industrial environment where the heat exchanger has been used—elements such as tanks, motors, valves, pipes, the heat exchanger itself, etc. Figure 7b presents the control area where three panels are implemented: the first is the control panel where the control variables (CV) are displayed, which are process temperature inputs, desired temperature values (SP), and disturbances such as mass flow rate (dm); the second monitor displays the hot outlet temperature $\left(Th_{o}\right)$; while the third monitor is used to display the variable cold outlet temperature $\left(Tc_{o}\right)$. This tool has become an aid in learning industrial process control with advanced control techniques.

### 4.2. Performance of the Proposed Controllers for the Countercurrent Plate Heat Exchanger

The two designed control strategies are compared. For the control based on the inverse of the plant, the diagonal weight matrix W with values of 0.2 on its diagonal is defined. For the MPC control, the parameters used for the plate heat exchanger and countercurrent flow are $\Delta u_{1\min}=0$ and $\Delta u_{1\max}=100$, and for the second control action, $\Delta u_{2\min}=0$ and $\Delta u_{2\max}=100$, which corresponds to the valve openings. The limits for the hot outlet temperature are $Th_{o\min}=0$ and $Th_{o\max}=75$, and for the cold outlet temperature, $Tc_{o\min}=0$ and $Tc_{o\max}=45$. These values represent the operating range of the exchanger. Regarding the tuning parameters of this controller, they are obtained heuristically by obtaining the weights of the hot outlet temperatures: $\gamma_{1}=1$ and $\gamma_{2}=1$, respectively. As for the weights for the control actions, $\rho_{1}=0.001$ and $\rho_{2}=0.001$ are defined. Then, we need additional parameters such as the prediction horizon $N_{f}=10$ in addition to the control horizon $N_{c}=1$ with a sampling time of 0.1 s.

**Figure 8:**
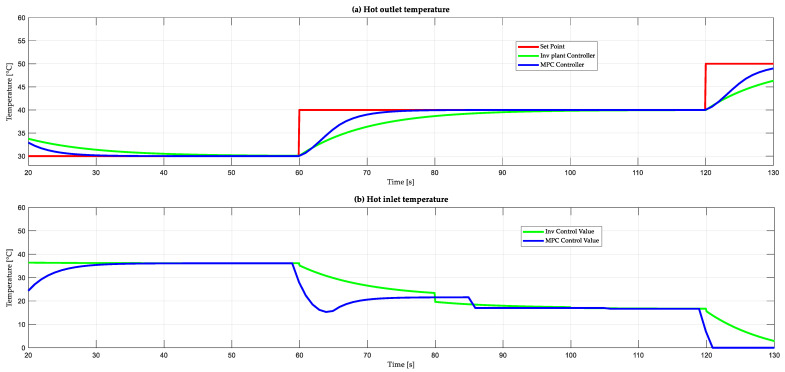
Controller performance based on plant inverse and MPC controller: (**a**) response of hot outlet temperature and (**b**) hot entry temperature.

Figure 8a shows the behavior of the hot outlet temperature under different control strategies. Control based on the inverse of the plant is represented in green, while MPC control is shown in blue. These strategies use a setpoint that varies every 60 s, the (SP) starts with a value of 30 °C, then changes to 40 °C, and finally changes to 50 °C. When analyzing the interval from 60 s to 120 s, neither controller presents overdrive. The MPC controller presents a lower settling time of 85 s compared to the controller based on the inverse of the plant which is 110 s. The MPC controller does not present steady-state error while the inverse model is minimal. With respect to the control actions in Figure 8b, the manipulated variable corresponding to the hot entry temperature is observed. For the case of the control based on the inverse of the plant, its control action begins to act from the instant of 0 s and reaches its steady state at the instant of 35 s, with a value of hot entry temperature of 36.19 °C. On the other hand, in the MPC control, its control action begins with a time of 5 s for its action and comes to stabilize at 36 s with a temperature value of 35.99 °C in equilibrium, showing that the hot inlet temperature is set in both controls at a value of approximately 36.00 °C to obtain the desired hot outlet temperature.

Table 1 presents the results of the control performance parameters of the implemented algorithms for the hot outlet temperature control. The overshoot, steady-state error, and settling time of the two controllers, based on the plant inverse and MPC, are compared. These parameters were analyzed in the plate heat exchanger and countercurrent flow. According to the values obtained, it is shown that the MPC control presents a lower settling time and lower steady-state error.

**Figure 9:**
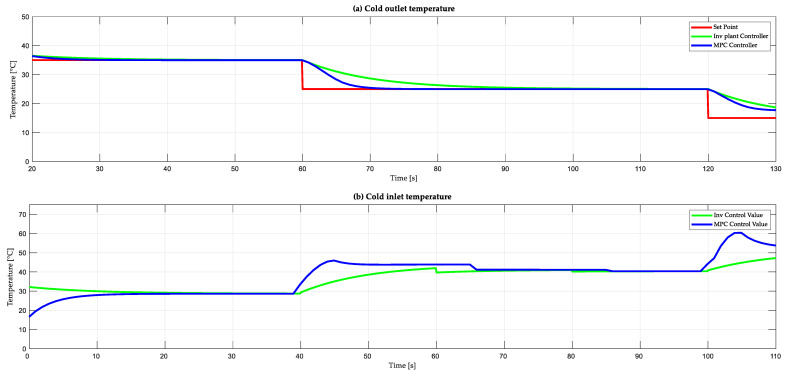
Controller performance based on plant inverse and MPC controller: (**a**) output cold temperature response and (**b**) input cold temperature.

Figure 9a shows the behavior of the output cold temperature against different control strategies: plant inverse-based control (green), and an MPC control (blue). The set point (red) varies every 60 s. The set point (SP) starts with a value of 35 °C, then changes to 25 °C, and finally changes to 15 °C. When analyzing the interval from 60 s to 120 s, neither controller presents overshoot. The MPC controller presents a lower settling time of 78 s compared to the controller based on the inverse of the plant which is 100 s. The MPC controller does not present steady-state error, while for the inverse model it is minimal. On the other hand, the control actions in Figure 9b show the manipulated variable corresponding to the input cold temperature. For the case of the control based on the inverse of the plant, its control action begins to act from the instant of 0 s reaches its stationary state at the instant of 40 s, with an inlet cold temperature value of 29.15 °C. On the other hand, the MPC control initiates its action at 12 s and stabilizes at 38 s with a temperature value of 28.63 °C. It can be observed that, in equilibrium, the cold inlet temperature is set to approximately 28.5 °C in both controls to achieve the desired hot outlet temperature.

Table 2 shows the control parameters such as the overshoot, time to enter steady state, and steady-state error of the two control algorithms based on the plant inverse and MPC for the control of the cold outlet temperature. It can be seen that the MPC control has a better performance compared to the control based on the inverse of the plant because the control actions are not abrupt. Consequently, the life of the actuator is taken care of and comes to stabilize faster than the other controller.

**Figure 10:**
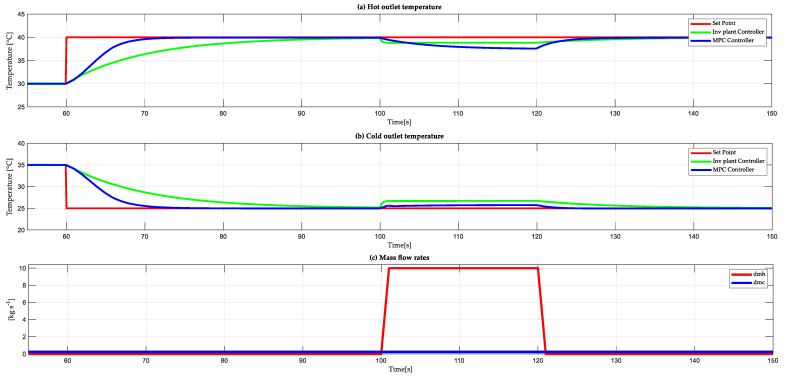
(**a**) Hot outlet temperature. (**b**) Cold outlet temperature. (**c**) Disturbances corresponding to the hot and cold mass flows $dm_{h}$.

The system is evaluated against external disturbances in order to test its robustness. Two perturbations are added by means of mass flows. Figure 10 shows the behavior of the system against perturbations. As shown in Figure 4, the external disturbances can be produced by changing the hot and cold mass flow $\left(dm_{h}\right)$ and $\left(dm_{c}\right)$, respectively. These add to the variables to be controlled and cause changes in the outlet temperatures to be controlled. At 100 s, a disturbance is caused by changing the hot mass flow $\left(dm_{h}\right)$, which causes a change in the temperatures to be controlled. The cold mass flow $\left(dm_{c}\right)$ has a constant value of 0.24 kg/s, while the hot mass flow $\left(dm_{h}\right)$ has a value of 10 kg/s in the range 100 s to 120 s. This perturbation has an effect on the whole system as the mass flow is related to the outlet temperatures (see Figure 10c). The system is perturbed with a hot mass flow of 10 kg/s at a time of 100 s (see Figure 10a). The hot outlet temperature when controlled with an MPC algorithm has a 10 s less settling time to compensate for the disturbance than control based on the plant inverse. Similarly, as shown in Figure 10b, the cold outlet temperature responds 21 s faster with the MPC control than with the control based on the plant inverse. As can be seen, the MPC controller performs better against perturbations.

The results of this research are compared with results shown in related works, such as the one reported in [18] where a comparison of controls for plate heat exchangers and countercurrent flow is shown. Conventional PI and PID controls are evaluated with an advanced control based on fuzzy logic which shows a better performance. These three control strategies were analyzed and compared with the results obtained in this research, and it can be determined that the MPC control reduces the stability time by 115 s. In addition, the control signals are cleaner and smoother.

## 5. Conclusions

Learning and professional training in industry are strengthened by the use of real industrial plant models in immersive virtual environments, due to their dynamics and the variables to be controlled resembling the mode of operation of a real industrial process. The virtualized countercurrent flow and plate heat exchanger has proven to be an immersive industrial process that enables the scope of different applications of linear, non-linear, and advanced control strategies. The communication link between the virtual environment and industrial process control variables is developed on a DLL platform, specifically designed for this type of teaching in a professional and educational method.

Two control algorithms for a virtualized countercurrent flow plate heat exchanger are implemented and compared: one based on the plant inverse and one MPC. Both controls present an acceptable performance with respect to the controlled variables of the hot outlet temperature and cold outlet temperature of the heat exchanger. However, the MPC control presents a better performance in a transient and a steady state. Both controllers show no overshoot values. The MPC control presents a reduction in the stability time of 35 s compared to the plant inverse-based controller. Also, in the case of the MPC control, there is no steady-state error, while in the inverse plant control, the error is 0.05%.

## Figures and Tables

**Table 1:** Performance of control algorithms for hot temperature output.

Parameters	Inverse-Based Control of the Plant	MPC Control
Overshoot [%]	0	0
Stability time [s]	110	85
Steady-state error	$1.75\times 10^{-3}$	$5\times 10^{-2}$

**Table 2:** Performance of control algorithms for cold temperature output.

Parameters	Inverse-Based Control of the Plant	MPC Control
Overshoot [%]	0	0
Stability time [s]	100	78
Steady-state error	$7.2\times 10^{-3}$	$7\times 10^{-2}$

## Data Availability

Data are contained within the article.
